# Transplantation of a single kidney from pediatric donors less than 10 kg to children with poor access to transplantation: a two-year outcome analysis

**DOI:** 10.1186/s12882-020-01895-6

**Published:** 2020-07-02

**Authors:** Xiaojun Su, Wenjun Shang, Longshan Liu, Jun Li, Qian Fu, Yonghua Feng, Huanxi Zhang, Ronghai Deng, Chenglin Wu, Zhigang Wang, Xinlu Pang, Björn Nashan, Guiwen Feng, Changxi Wang

**Affiliations:** 1 Organ Transplant Center, The First Affiliated Hospital of Sun Yat-sen University, 58 Zhongshan 2nd Road, Guangzhou, 510080 People’s Republic of China; 2 Kidney Transplant Center, The First Affiliated Hospital of Zhengzhou University, 1 Construction Road, Zhengzhou, 450052 People’s Republic of China; 3 Organ Transplant Center, The First Affiliated Hospital of University of Science and Technology of China, Hefei, 230027 People’s Republic of China; 4 Guangdong Provincial Key Laboratory on Organ Donation and Transplant Immunology, Guangzhou, People’s Republic of China

**Keywords:** Pediatric donor, Single kidney transplantation, Pediatric transplantation, Outcomes

## Abstract

**Background:**

Access to kidney transplantation by uremic children is very limited due to the lack of donors in many countries. We sought to explore small pediatric kidney donors as a strategy to provide transplant opportunities for uremic children.

**Methods:**

A total of 56 cases of single pediatric kidney transplantation and 26 cases of *en bloc* kidney transplantation from pediatric donors with body weight (BW) less than 10 kg were performed in two transplant centers in China and the transplant outcomes were retrospectively analyzed.

**Results:**

The 1-year and 2-year death-censored graft survival in the *en bloc* kidney transplantation (KTx) group was inferior to that in the single KTx group. Subgroup analysis of the single KTx group found that the 1-year and 2-year death-censored graft survival in the group where the donor BW was between 5 and 10 kg was 97.7 and 90.0%, respectively. However, graft survival was significantly decreased when donor BW was ≤5 kg (*p* < 0.01), mainly because of the higher rate of thrombosis (*p* = 0.035). In the single KTx group, the graft length was increased from 6.7 cm at day 7 to 10.5 cm at 36 months posttransplant. The estimated glomerular filtration rate increased up to 24 months posttransplant. Delayed graft function and urethral complications were more common in the group with BW was ≤5 kg.

**Conclusions:**

Our study suggests that single kidney transplantation from donors weighing over 5 kg to pediatric recipients is a feasible option for children with poor access to transplantation.

## Background

The incidence of end-stage renal disease (ESRD) in children has increased to 14 per million population (PMP), accounting for approximately 4.0% of all ESRD patients [1]. Long waiting time on dialysis is associated with significant morbidity and mortality [2]. In comparison with dialysis, kidney transplantation (KTx) provides a better quality of life and superior physical and psychological development for children with ESRD [3, 4]. Unfortunately, access to KTx by children with ESRD is limited because of the scarcity of donor kidneys in many developing countries. It was reported that only 10% children received dialysis and less than 1% received KTx in Pakistan [5]. The incidence of pediatric KTx is 0.87 PMP in some developing countries, while it is 5–10 PMP in developed countries [6]. As we previously reported, only 851 pediatric KTx were performed in the past 30 years in China, accounting for fewer than 2% of total KTx during the same period [7]. Living donations from parents are decreasing in recent years because of socioeconomic problems and medical considerations. It is urgent to find a new strategy to expand the organ donor pool and to improve children’s access to kidney transplantation, especially in developing countries.

It has been reported that kidneys from deceased pediatric donors provide outcomes comparable or even better outcomes than those from adult deceased donors [8, 9]. There has been a long and intense discussion around these two kinds of deceased kidneys in the past [10]. Following these discussions, particularly in response to findings related to the growth and development in pediatric recipients, allocation policies were changed, and pediatric donor kidneys are often allocated preferentially to pediatric recipients. These policies include those of Eurotransplant from March 2019 and the National Health Service Blood and Transplant (NHSBT). Pediatric kidneys are usually transplanted *en bloc* into adult or pediatric recipients [11–17]. While satisfactory long-term graft function has been achieved, *en bloc* transplantation of small kidneys may result in a high risk of early graft loss mainly due to vascular thrombosis [18]. This hinders the utilization of kidneys from small deceased donors and leads to a high discard rate of up to 40.3% when the donor’s body weight (BW) is less than 10 kg [19]. On the other hand, single kidney transplantation from small pediatric donors to pediatric recipients (P to P) has resulted in favorable outcomes [20–22]. This strategy can not only expand the potential pediatric donor pool but also provide more transplant opportunities for uremic children. Regarding the utilization of small kidneys from pediatric donors with body weight less than 10 kg, we have performed many cases of *en bloc* and single kidney transplantation. In this retrospective study, we compared our two-year outcome data of these two techniques to provide evidence regarding the safety of single transplantation of small pediatric donor kidneys, and most importantly to further determine the suitable donor body weight for single transplantation from pediatric donors to pediatric recipients by subgroup analysis of the single kidney transplantation P to P cohort data.

## Methods

### Study design

From May 2014 to April 2018, all single kidney transplantations in children and *en bloc* kidney transplantations from pediatric donors whose BW was below or equal to 10 kg performed at the First Affiliated Hospital of Sun Yat-sen University and the First Affiliated Hospital of Zhengzhou University were retrospectively analyzed. All kidneys were from deceased pediatric donors of one of three different donor types: donation after brain death (DBD), donation after cardiac death (DCD) and donation after brain and cardiac death (DBCD). DBCD means the donor was qualified as a DBD donor, but his/her family decided to complete the donation only after the patient suffered circulatory death. The donation was completed in strict accordance with DCD [23, 24]. There were 56 cases of single kidney transplantation in children and 26 cases of *en bloc* kidney transplantation in adults and children. Patient and graft survival of *en bloc* KTx and single KTx were compared to determine the safety of P to P single KTx. Further subgrouping the single-KTx P to P cohort into two subgroups based on donor BW was done to analyze the suitable donor BW for single KTx in pediatric recipients. Therefore, these 56 single KTx cases were divided into two groups: 13 KTx from donors with BW ≤5 kg and 43 KTx from donors with BW between 5 and 10 kg. This grouping strategy was based on the finding that *en bloc* KTx from donors ≤5 kg and donors > 5 kg resulted in similar graft survival [15]. Informed consent of each donor’s family for organ donation was obtained before donation. This research upheld the principles of the Declaration of Istanbul as outlined in the “Declaration of Istanbul on Organ Trafficking and Transplant Tourism”. This study was approved by the institutional ethics committees.

### Surgical technique and perioperative care

*En bloc* kidneys were recovered with the aorta, vena cava and bilateral ureters. When considering *en bloc* kidney transplantation, the abdominal aorta and inferior vena cava were used for anastomosis. The distal ends of the aorta and vena cava were closed inferior to the renal vessels, while the proximal ends of the donor aorta and vena cava were anastomosed to the external iliac artery and vein in an end-to-side manner using 6–0 or 7–0 prolene. The *en bloc* graft was placed properly to prevent vessel distortion in the iliac fossa. Two ureteroneocystostomies were performed separately by the Lich-Gregoir technique with placement of ureteral stents. When considering single kidney transplantation, *en bloc* donor kidneys were split into two single kidneys on the back table. Tissues around the renal artery and renal vein were kept undissected to avoid vessel irritation. Vascular patches were made for anastomosis using the donor aorta. Single graft kidney was anastomosed to external iliac blood vessels in an end-to-side manner with a running suture using 6–0 prolene and was placed in the iliac fossa extraperitoneally. Ureteroneocystostomy was also performed with placement of a ureteral stent. To prevent vasospasm, papaverine (30 mg) was directly injected into the renal graft artery before blood reperfusion and was continuously pumped at 2 mL/h (60 mg in 50 mL of saline) for three days after transplantation.

Similar anticoagulation protocols were used in these two centers. In the First Affiliated Hospital of Sun Yat-sen University, all the recipients received low-molecular-weight heparin (LWMH, 50–100 IU/kg/d) for approximately one to five days, and minor adjustments of the dosage were made based on the postoperative drainage volume around the allograft and on graft ultrasound examination. LMWH was subsequently switched to oral antiplatelet medication, and then the oral antiplatelet medication was gradually discontinued. In the First Affiliated Hospital of Zhengzhou University, the basic principle of initiation of prophylactic anticoagulation therapy is when the donor age was younger than one year old or the recipient was younger than 5 years old. LMWH (50–100 IU/kg/d) was also used as anticoagulation therapy one to five days posttransplant, but the dosage was adjusted according to drainage volume and thromboelastography. Additionally, LMWH was subsequently switched to oral antiplatelet medication and then the oral antiplatelet medication was gradually discontinued.

The systolic blood pressure of the recipients was maintained below 120 mmHg. In these 56 single kidney transplantation cases, all recipients received thymoglobulin as induction therapy except for one recipient who received basiliximab. In these 26 *en bloc* kidney transplantation cases, 23 recipients received thymoglobulin as induction therapy and the other three received basiliximab. The maintenance immunosuppressive therapy consisted of tacrolimus, mycophenolate mofetil and corticosteroids.

### Transplant outcome parameters

The estimated glomerular filtration rate (eGFR) was calculated using the Schwartz formula for the assessment of renal graft function [25]. Graft ultrasound examination was consecutively implemented during follow-up, and the length of the kidneys was measured to determine graft growth. In the same hospital, one or two ultrasound specialists were responsible for renal allograft examination to ensure the accuracy of measurement and reduce subjectivity. All ultrasound doctors were blinded to the initial donor weight subgroup. Posttransplant complications including primary nonfunction (PNF), delayed graft function (DGF), vascular thrombosis, ureteral complications, rejection, infection and recurrence of primary diseases, were collected. Patient and graft survival were analyzed, and graft loss was defined as graft nephrectomy or irreversible return to dialysis.

### Statistical analysis

All measurement data are presented as median with quartiles or range. Categorical variables were analyzed with the χ^2^ test and Fisher’s exact test, while continuous variables were analyzed with the Mann-Whitney U test and Wilcoxon signed-rank test. The Kaplan-Meier method was used to determine patient and death-censored graft survival, and Breslow-Wilcoxon test and log rank test were used for subgroup analysis. Statistical significance was accepted when the *p* value was less than 0.05. All statistical analyses were conducted with SPSS version 21 (SPSS Inc., Chicago, IL) and GraphPad Prism version 7.0 for Windows (GraphPad Software, La Jolla, CA).

## Results

### Demographic characteristics

All recipients in the *en bloc* KTx group received their first kidney graft, while two recipients in the single KTx group received their second kidney graft, the others their first. The donor and recipient characteristics of these two transplant types were summarized in Table S1. Fourteen patients receiving *en bloc* KTx were adults, accounting for 54% of the total *en bloc* KTx. The percentages of DBD, DCD and DBCD were similar between these 2 groups (28.1, 59.4 and 12.5% in single KTx versus 19.2, 69.2 and 11.6% in *en bloc* KTx, *p* > 0.05).

The median BW of donors in the single KTx group was similar to that in the *en bloc* KTx (7.8 kg vs 6.7 kg, *p* > 0.05), but the median BW of recipients in the single KTx was lower than that in the *en bloc* KTx group (22.0 kg vs 37.75 kg, *p* < 0.001). Therefore, the donor/recipient BW ratio was much higher in *en bloc* KTx (median 1:7.9 vs 1:3.2, *p* < 0.001).

The donor and recipient characteristics of the single KTx group were summarized in Table 1. The median age of donors was 8.9 months, with a range from 10 days to 3.4 years old. The median BW of donors was 7.8 kg, with a range from 3.0 to 10 kg. The median age and BW of recipients were 9.0 years old and 22.0 kg, respectively. Preemptive transplantations were performed in 6 patients. The median follow-up time was 23 months, with a range of 1 days to 53 months. When the study population was divided into two subgroups based on donor weight (BW ≤ 5 kg vs 5 < BW ≤ 10 kg), only donor age, donor weight and donor/recipient body weight ratio were significantly different between the BW subgroups. The donor age and donor weight were significantly lower in the BW ≤ 5 kg group, while the donor/recipient BW ratio was significantly lower in the 5 < BW ≤ 10 kg group.

**Table 1:** Clinical characteristics of pediatric donors and recipients in single kidney transplantation group

Donor Characteristics	Overall (*n* = 32)	BW ≤ 5 kg (*n* = 7)	5 < BW ≤ 10 kg (*n* = 25)
Age, month (Range)*	8.9 (0.3–41.3)	1.3 (0.3–5.6)	12.0 (1.7–41.3)
Sex, male/female (%)	15 (46.9%)/17 (53.1%)	2 (28.6%)/5 (71.4%)	13 (52.0%)/12 (48.0%)
Body weight, kg (Range)*	7.8 (3.0–10.0)	3.9 (3.0–5.0)	9.0 (5.2–10.0)
Causes of death, number (%)			
Brain injury	7 (21.9%)	0	7 (28.0%)
Pneumonia or respiratory failure	6 (18.8%)	3 (42.9%)	3 (12.0%)
Cerebral hemorrhage or hernia	5 (15.6%)	1 (14.3%)	4 (16.0%)
Intracranial infection	3 (9.4%)	1 (14.3%)	2 (8.0%)
Brain tumor	2 (6.3%)	0	2 (8.0%)
Trauma	2 (6.3%)	0	2 (8.0%)
Congenital heart disease	1 (3.1%)	1 (14.3%)	0
Unknown	6 (18.8%)	1 (14.3%)	5 (20.0%)
Warm ischemia time, min (Range)	5.0 (0.0–12.0)	5.0 (0.0–5.0)	5.0 (0.0–12.0)
Cold ischemia time, hour (Range)	8.0 (2.0–23.5)	10.0 (6.0–22.0)	7.0 (2.0–23.5)
Recipient characteristics	Overall (*n* = 56)	BW ≤ 5 kg (*n* = 13)	5 < BW ≤ 10 kg (*n* = 43)
Age, year	9.0 (1.4–17.0)	7.0 (5.0–12.0)	9.0 (1.4–17.0)
Sex, male/female (%)	32 (57.1%)/24 (42.9%)	9 (69.2%)/4 (30.8%)	25 (58.1%)/18 (41.9%)
Body weight, kg (Range)	22.0 (6.5–44.0)	23.0 (13.0–29.0)	22.0 (6.5–44.0)
Donor/Recipient BW Ratio, (Range)*	1:3.2 (1:0.7–1:9.1)	1:5.6 (1:2.6–1:9.1)	1:2.6 (1:0.7–1:6.25)
Waiting time since dialysis, month (Range)	8.0 (0.0–72.0)	7.0 (0.0–36.0)	8.0 (0.0–72.0)
Type of dialysis, number (%)			
Preemptive	6 (10.7%)	1 (7.7%)	5 (11.6%)
PD	20 (35.7%)	3 (23.1%)	17 (39.5%)
HD	25 (44.7%)	7 (53.8%)	18 (41.9%)
PD + HD	5 (8.9%)	2 (15.4%)	3 (7.0%)
HLA mismatch number (Range)	3 (2–6)	3 (3–6)	3 (2–6)
Primary disease, number (%)			
Glomerulonephritis	35 (62.5%)	10 (76.9%)	25 (58.1%)
FSGS	5 (8.9%)	1 (7.7%)	4 (9.3%)
IgA nephropathy	3 (5.3%)	0	3 (7.0%)
Congenital renal dysplasia	2 (3.6%)	1 (7.7%)	1 (2.3%)
Other	11 (19.7%)	1 (7.7%)	10 (23.3%)
Follow-up time, month (Range)	23.0 (0.03–53.0)	26.0 (0.03–34.6)	21.5 (0.3–53.0)

*BW* body weight, *FSGS* focal segmental glomerulosclerosis, *HD* hemodialysis, *PD* peritoneal dialysis

* *p* < 0.05 for BW ≤ 5 kg vs 5 < BW ≤ 10 kg comparisons

### Patient and graft survival

**Fig. 1:**
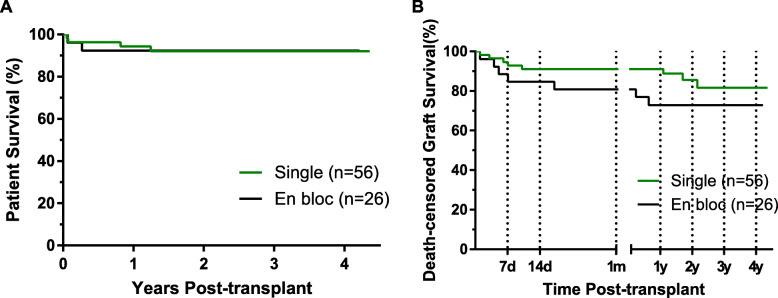
Patient and graft survival in the single and *en bloc* kidney transplantation groups. **a.** Patient survival. There were no significant differences between the two groups. **b.** Death-censored graft survival. The death-censored graft survival in the *en bloc* kidney transplantation group was inferior to that in the single kidney transplantation group

The 1-year and 2-year patient survival rates were 92.3 and 92.3% in the *en bloc* KTx group, respectively, which was similar to those of the single KTx group (Fig. 1a). A total of seven graft losses occurred in the *en bloc* KTx group, four of which were lost within 7 days. The major reason for graft loss in the *en bloc* KTx group was thrombosis, accounting for 57.1%. The reasons for graft loss in the remaining 3 cases included PNF, persistent urinary leak and acute rejection due to discontinuation of immunosuppressive drugs when treating *Pneumocystis carinii* pneumonia. Therefore, the 1-year and 2-year death-censored graft survival of the *en bloc* KTx group was 72.9 and 72.9%, respectively, which were inferior to those of the single KTx group (Fig. 1b), indicating that the P to P single-KTx strategy could provide superior graft survival for ESRD children.

**Fig. 2:**
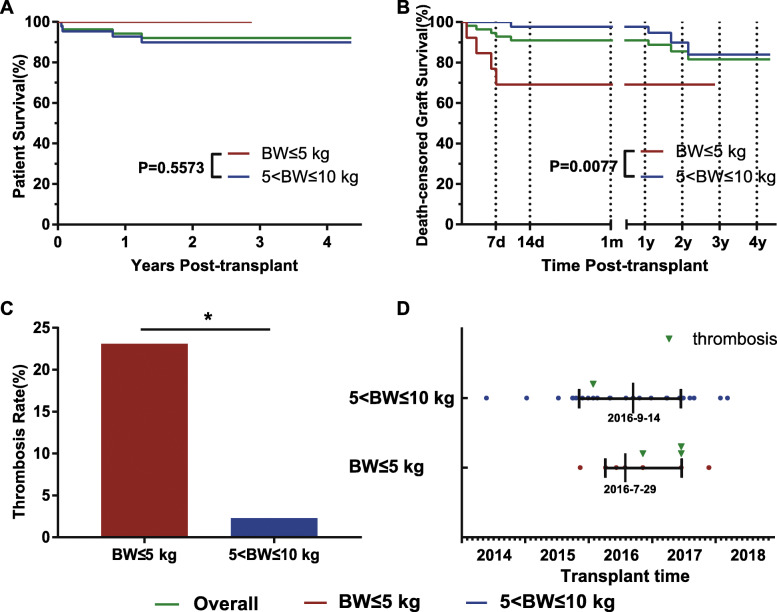
Patient and graft survival in pediatric kidney transplantation from pediatric donors (BW ≤ 10 kg). **a.** Patient survival. No patients in the BW ≤ 5 kg group died, while there were 4 deaths in children with functional grafts in the 5 < BW ≤ 10 kg group, but there was no significant difference between the two groups; **b.** Death-censored graft survival. A significant difference was found between the two groups (*p* = 0.0077); **c.** Vascular thrombosis rate (*p* = 0.035 for BW ≤ 5 kg vs 5 < BW ≤ 10 kg comparison); **d.** Distribution of transplant date. Similar transplant date distributions between the two groups exclude learning-curve effects on the utilization of different body weight donors. BW, body weight

We further analyzed the P to P single KTx group and divided it into two groups based on donor BW. The overall 1-year and 2-year patient survival rates were 94.3 and 92.1%, respectively, and the 1-year and 2-year death-censored graft survival rates were 91.1 and 85.5%, respectively (Fig. 2a, b). Subgroup analysis found that death-censored graft survival dramatically decreased when donor body weight was below or equal to 5 kg (BW ≤ 5 kg vs 5 kg < BW ≤ 10 kg, *p* = 0.0077) (Fig. 2b). In the 5 kg < BW ≤ 10 kg group, the 1-year and 2- year death-censored graft survival rates were 97.7 and 90.0%, respectively. Two grafts lost because of chronic rejection at 13 months and 21 months after transplantation.

Notably, four grafts were lost within 7 days after transplantation in the BW ≤ 5 kg group (Fig. 2b), and three of them were due to vascular thrombosis, leading to a rapid drop in graft survival to 69.2% at day 7. Only one thrombosis occurred in the 5 kg < BW ≤ 10 kg group. The rate of vascular thrombosis was significantly different between the two groups as shown in Fig. 2c (23.1% vs 2.3%, *p* = 0.035). With regard to transplant date, there was no difference between the BW ≤ 5 kg group and 5 kg < BW ≤ 10 kg group (*p* = 0.719), and there was no difference between patients with thrombosis and those without thrombosis within the BW ≤ 5 kg group (*p* = 0.161) (Fig. 2d). These findings may exclude learning curve effects on the risk of thrombosis occurrence.

Unlike the *en bloc* KTx group, recipients in the single KTx group could receive kidney grafts from the same donor, and 48 recipients received kidney grafts from 24 donors in our study. The graft outcomes of these 48 recipients were summarized in Table 2. Five of these 48 recipients lost their kidney grafts, and four of them received kidneys from two donors weighing ≤5 kg (Table 2, Case 1 and Case 2), while the remaining one received a kidney from a donor weighing over 5 kg (Table 2, Case 3).

**Table 2:** Fates of kidneys from the same donor in the single kidney transplantation group

Donor	Donor Weight	Recipient	Graft status	Cause of graft failure
Case 1	≤5 kg	1	Fail	thrombosis
		2	Fail	thrombosis
Case 2	≤5 kg	1	Fail	Thrombosis
		2	Fail	PNF
Case 3	5–10 kg	1	Fail	Recurrence of IgA nephropathy
		2	Function	–
Case 4–7	≤5 kg	–	Function	–
Case 8–24	5–10 kg	–	Function	–

*PNF* primary nonfunction

### Renal growth and graft function in the single KTx group

**Fig. 3:**
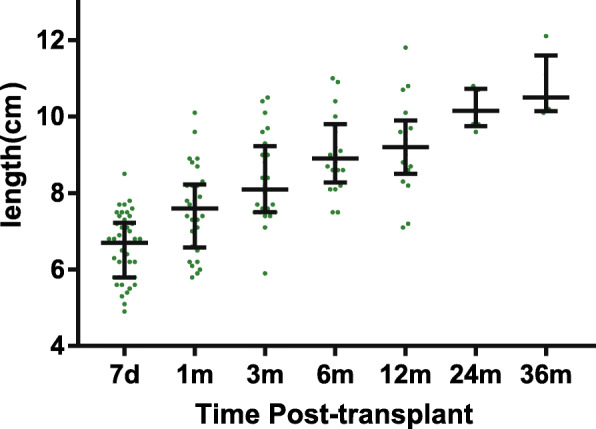
Dynamic change in renal graft length after kidney transplantation. Renal grafts presented adaptive growth after kidney transplantation. Note that graft length presented increasing tendency even until 36 months posttransplant

**Fig. 4:**
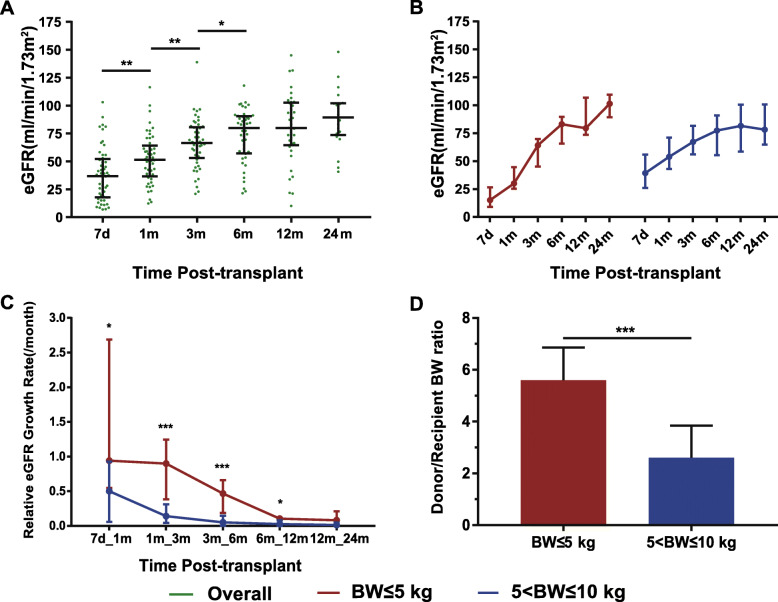
Dynamic change in renal graft function after kidney transplantation. **a.** eGFR level in 56 patients (comparison between different time: 7 days vs 1 month, *p* = 0.002; 1 month vs 3 months, p = 0.002; 3 months vs 6 months, *p* = 0.043); **b.** eGFR in the BW ≤ 5 kg and 5 < BW ≤ 10 kg groups. A similar increasing tendency was seen in both groups; **c.** Relative eGFR growth rate in the BW ≤ 5 kg and 5 < BW ≤ 10 kg groups. Different relative eGFR growth rates were observed in the first year after transplantation. BW ≤ 5 kg vs 5 < BW ≤ 10 kg comparisons: 7 days_1 month: *p* = 0.034, 1 month_3 months: *p* < 0.001, 3 months_6 months: *p* < 0.001, 6 months_12 months: *p* = 0.019); **d.** Donor/recipient body weight ratio (*p* < 0.001 for BW ≤ 5 kg vs 5 < BW ≤ 10 kg comparison). BW, body weight; eGFR, estimated glomerular filtration rate

The length of the renal graft in the single KTx group after transplantation was shown in Fig. 3. Thirteen kidneys were less than 6 cm in length at day 7, among which 7 were from BW ≤ 5 kg donors. Notably, graft length steadily increased from 6.7 cm at day 7 to 10.5 cm at 36 months posttransplant. Renal graft function was shown in Fig. 4. The eGFR dramatically increased within the first 6 months after transplantation from 36.8 ml/min/1.73m^2^ to 79.9 ml/min/1.73m^2^ (7 days vs 1 month, *p* = 0.002; 1 month vs 3 months, *p* = 0.002; 3 months vs 6 months, *p* = 0.043) and still presented an increasing tendency even up to 24 months posttransplant, reaching a median eGFR of 89.4 ml/min/1.73m^2^ (Fig. 4a). These 56 patients were then divided into two groups as shown in Fig. 4b and c. The subgroup analysis found that eGFR in both groups significantly increased (Fig. 4b), but the relative growth rate of eGFR was significantly higher in BW ≤ 5 kg group (Fig. 4c). The body weight ratio of donor to recipient was significantly higher in BW ≤ 5 kg group (Fig. 4d), which may explain this group’s higher growth rate of eGFR.

### Complications in the single KTx group

Posttransplant complications in the single KTx group were summarized in Table 3. The most common complication was infection (23.2%), among which pulmonary infections predominated (11/13, 84.6%). Three out of 11 pulmonary infections led to patient death. Seven episodes of delayed graft function (DGF) occurred. Six of them recovered to normal graft function, while one ended with graft loss due to late vascular thrombosis at day 9. The DGF rate was significantly higher in the BW ≤ 5 kg group (30.8% vs 12.5%, *p* = 0.043). As mentioned above, four cases of vascular thrombosis occurred, though all of these patients had received prophylactic anticoagulation therapy, and all led to early graft loss. Ureteral complications including three stenoses and one urine leakage, were observed. Most of them occurred in the BW ≤ 5 kg group, and all were cured after conservative or surgical treatment. Five biopsy-proven rejections were observed, two of which led to late graft loss. One recurrent IgA nephropathy resulted in late graft loss.

**Table 3:** Posttransplant complications in pediatric recipients of the single kidney transplantation group

Complications	Overall (%) (n = 56)	BW ≤ 5 kg (%) (n = 13)	5 < BW ≤ 10 kg(%) (n = 43)
Infection	13 (23.2%)	1 (7.7%)	12 (25.6%)
Delayed graft function*	7 (12.5%)	4 (30.8%)	3 (7.0%)
Recurrence of primary disease	6 (10.7%)	1 (7.7%)	5 (11.6%)
Biopsy-proven rejection	5 (8.9%)	0	5 (11.6%)
Vascular thrombosis*	4 (7.1%)	3 (23.1%)	1 (2.3%)
Ureteral complication*	4 (7.1%)	3 (23.1%)	1 (2.3%)
Primary nonfunction	1 (1.8%)	1 (7.7%)	0

*BW* body weight

* *p* < 0.05 for BW ≤ 5 kg vs 5 < BW ≤ 10 kg comparisons

## Discussion

There are many challenges in developing a pediatric kidney transplant program in many countries. One of the major obstacles is the limited access to high-quality donor kidneys. Unsatisfactory dialysis treatment causes high morbidity and mortality in uremic children, especially small children. On the other hand, the high incidence of vascular thrombosis hampers *en bloc* transplantation of small kidneys, especially when donor body weight is less than 10 kg. A higher vascular thrombosis rate and lower death-censored graft survival were observed in the *en bloc* KTx group in this study. The results of this study indicate that successful transplantation can be obtained by implementing the P to P single-kidney-transplant strategy when donor body weight was over 5 kg. This technique has the potential not only to improve the effective utilization of small pediatric donor kidneys but also, more importantly, to provide uremic children with an opportunity to receive a kidney transplantation. To our knowledge, this is the largest retrospective analysis of single kidney transplantation from pediatric donors (BW ≤ 10 kg) to pediatric recipients.

*En bloc* kidney transplantation with small pediatric kidneys into one adult patient was thought to be a relatively safe way to offer superior graft survival because of enough nephron mass. However, such incremental benefits to an individual adult come at the substantial expense of penalizing two waitlist children. Maluf et al. [26] demonstrated that a positive net gain could be achieved if all donated kidneys, even from donors weighing less than 10 kg, were performed in a single-kidney-transplant technique. In addition, compared to *en bloc* kidney transplantation into adult recipients, single pediatric transplantation has the following merits. (1) It can benefit twice as many pediatric recipients. (2) The surgical technique is similar to the conventional procedure, so a long learning curve is not necessary to achieve standardization. (3) It is more convenient to place one renal graft into the iliac fossa, and this can significantly reduce the risk of vascular torsion [27]. Indeed, thrombosis has been a major deterrent for the utilization of extremely low-body-weight donor kidneys. The reported rate of vascular thrombosis is as high as 9.1–25% in *en bloc* kidney transplantation from pediatric donors weighing less than 10 kg [13, 14, 16, 17]. In this study, the vascular thrombosis rate was higher (15.4%) in the *en bloc* KTx group, but it was dramatically lower (2.3%) when donor body weight was 5–10 kg in the single KTx group. This result reflects the surgical advantages of single kidney transplantation from small pediatric donors. In addition, meticulous operation avoiding vascular irritation during the whole procedure from organ recovery to transplantation, and peri-operative papaverine administration are also very important to reduce vasospasm and thrombosis [16]. Despite these efforts, the thrombosis rate was still very high (23.1%) when donor body weight was below 5 kg, which led to significant lower graft survival. Our analysis of the transplant date excluded a learning-curve effect. This result reminds us to cautiously utilize of very small kidneys from donors weighing less than 5 kg. It was recently reported that eight *en bloc* transplantations of kidneys from infant donors weighing 1.9–4.9 kg were successful by constructing an additional blood outflow tract [28]. This technique may provide a new way to improve the effective utilization of very small donor kidneys.

DGF is another common complication after kidney transplantation from small pediatric donors. It was reported that 19.2% of recipients developed DGF when receiving kidneys from pediatric donors weighing less than 10 kg [15], and the DGF rate increased to 45.5% when patients were adult recipients of *en bloc* transplantation [13]. Unlike acute injuries in adult deceased donor kidneys, the main cause of DGF in small pediatric donor kidneys was insufficient functional nephrons at the early phase after transplantation [29]. Matching of pediatric donors to pediatric recipients by body weight can reduce DGF occurrence. Indeed, the DGF rate was only 12.5% in this study. More importantly, subgroup analysis showed a significantly lower rate of DGF (7.0%) when donor body weight was over 5 kg. This low DGF might be mainly attributed to more suitable body weight matching as shown in Fig. 4d.

Transplantation in children serves to restore their potential for normal growth and development. A graft therefore must provide good renal function to allow adequate growth [8]. Living donated kidneys from their parents provide superior graft outcomes, but living donations have decreased in recent years. Suitable parent living donations do not always happen because of socioeconomic problems, medical considerations and religious beliefs [30]. On the other hand, with the increase of pediatric deceased donation, suitable donor-recipient matching can be acquired by utilizing pediatric kidneys. Moreover, primary deceased-donor KTx followed by secondary living-donor KTx provides a similar cumulative graft life as primary living-donor KTx followed by secondary deceased-donor KTx for pediatric recipients [31]. This finding suggests that living-related donation for children could be saved for secondary KTx when necessary. Adult donor kidneys with sufficient nephron mass are commonly considered as favorable choices. However, a recent study argued that adult kidneys lack the capacity to increase their function according to subsequent body growth in children [32]. Our study demonstrated that renal grafts from small pediatric donors gained adaptive growth in pediatric recipients and that eGFR gradually increased to meet their metabolic demand. These findings are consistent with previous studies that demonstrated the great growth potential of pediatric renal grafts in both size [33] and function [32] with the physical development of pediatric recipients.

Children have nationwide priority for allocation of pediatric donors in many developed countries [34–37]. These policies shorten the waitlist time and may facilitate long-term transplant outcomes. However, children in the United States have not benefitted from priorities for high-quality pediatric donors or shortened waitlist time after the implementation of a new kidney allocation system, which stirred much controversy [38]. Pediatric deceased donation has significantly promoted the development of the pediatric KTx program within recent years in China, and new regulations on organ sharing have announced the nationwide priority of pediatric donor organs for children since August 2018. More pediatric donor kidneys would be transplanted to pediatric recipients in the single-transplant technique, and its long-term outcomes are worth exploring. In particular, this type of policy change has already helped considerably to improve the allocation for children within Eurotransplant [39].

Our study suffers from the limitations of a retrospective analysis. For instance, not enough cases could be recruited to determine the impact of donor types and cold ischemia time on short-term and long-term outcomes. In addition, data concerning posttransplant donor-specific antibodies and patient adherence were unavailable. We are thus unable to assess the potential negative impact of nonadherent adolescent recipients since some grafts were lost late due to chronic rejection. Another limitation of this study is the limited number of study population and lack of available long-term data. But this could be addressed by conducting a larger multicenter study and continuing to follow up the study cohort.

## Conclusions

In conclusion, single kidney transplantation from pediatric donors weighing 5–10 kg to pediatric recipients is feasible and has favorable outcomes. This strategy may help to improve access to kidney transplantation for children with poor access to transplantation in many countries. More studies with larger patient cohorts and longer follow-up periods are needed to substantiate our findings and move this field forward.

## Supplementary information

**Additional file 1: Table S1.** Clinical characteristics of donor and recipients in single and *en bloc* kidney transplantation group.

## Data Availability

The datasets used and analyzed during the current study are available from the corresponding author on reasonable request.

## Abbreviations

BW: Body weight
DBD: Donation after brain death
DBCD: Donation after brain and cardiac death
DCD: Donation after cardiac death
DGF: Delayed graft function
ESRD: End-stage renal disease
eGFR: Estimated glomerular filtration rate
FSGS: Focal segmental glomerulosclerosis
HD: Hemodialysis
KTx: Kidney transplantation
LMWH: Low molecular weight heparin
NHSBT: National health service blood and transplant
PD: Peritoneal dialysis
PMP: Per million population
PNF: Primary nonfunction
