# Supplementary material for: Transplantation of a single kidney from pediatric donors less than 10 kg to children with poor access to transplantation: a two-year outcome analysis
Source: BMC Nephrol. 2020 Jul 2;21:250. doi: 10.1186/s12882-020-01895-6 (PMC7330989; doi:10.1186/s12882-020-01895-6)
Supplement: Supplementary file 1 — Additional file 1: Table S1. Clinical characteristics of donor and recipients in single and en bloc kidney transplantation group. [file 12882_2020_1895_MOESM1_ESM.docx]

Table S1. Clinical characteristics of donor and recipients in single and *en bloc* kidney transplantation group.

| Donor Characteristics | Single  (n=32) | En bloc  (n=26) | P value |
| --- | --- | --- | --- |
| Age, month (Range) | 8.9 (0.3-41.3) | 4.75 (0.6-13.0) | 0.043 |
| Gender, male/female (%) | 15 (46.9%)/ 17 (53.1%) | 21 (80.8%)/ 5 (19.2%) | 0.008 |
| Body weight, kg (Range) | 7.8 (3.0-10.0) | 6.7 (2.65-10.0) | NS |
| Causes of death, number (%) | |  | NS |
| Brain injury | 7 (21.9%) | 1 (3.8) |  |
| Pneumonia or respiratory failure | 6 (18.8%) | 3 (11.5%) |  |
| Cerebral hemorrhage or hernia | 5 (15.6%) | 4 (15.4%) |  |
| Intracranial infection | 3 (9.4%) | 3 (11.5%) |  |
| Brain tumor | 2 (6.3%) | 2 (7.7%) |  |
| Trauma | 2 (6.3%) | 5 (19.2%) |  |
| Congenital heart disease | 1 (3.1%) | 3 (11.5%) |  |
| Unknown | 6 (18.8%) | 5 (19.5%) |  |
| Donor type, number (%) |  |  | NS |
| DBD | 9 (28.1%) | 5 (19.2%) |  |
| DCD | 19 (59.4%) | 18 (69.2%) |  |
| DBCD | 4 (12.5%) | 3 (11.5%) |  |
| Warm ischemia time, min (Range) | 5.0 (0.0-12.0) | 5.0 (0.0-30.0) | NS |
| Cold ischemia time, hour (Range) | 8.0 (2.0-23.5) | 12.0 (2.0-23.0) | 0.001 |
| Recipient characteristics | Single  (n=56) | En bloc  (n=26) | P value |
| Age, year (Range) | 9.0 (1.4-17.0) | 27.5 (7.3-51.6) | <0.001 |
| Gender, male/female (%) | 32 (42.9%)/24 (57.1%) | 10 (38.5%)/ 16 (61.5%) | NS |
| Body weight, kg (Range) | 22.0 (6.5-44.0) | 37.75 (20.5-81.0) | <0.001 |
| Donor/Recipient BW Ratio, (Range) | 1:3.2  (1:0.7-1:9.1) | 1:7.9  (1:2.6-1:15.0) | <0.001 |
| Waiting time since dialysis, month (Range) | 8.0 (0.0-72.0) | 15.9 (0.0-87.7) | 0.028 |
| Type of dialysis, number (%) | |  | NS |
| Preemptive | 6 (10.7%) | 1 (3.8%) |  |
| PD | 20 (35.7%) | 7 (26.9%) |  |
| HD | 25 (44.7%) | 18 (69.2%) |  |
| PD + HD | 5 (8.9%) | 0 |  |
| HLA mismatch number (Range) | 3 (2-6) | 3 (3-6) | NS |
| Primary disease, number (%) | |  |  |
| Glomerulonephritis | 35 (62.5%) | 13 (50.0%) | NS |
| FSGS | 5 (8.9%) | 0 | NS |
| IgA nephropathy | 3 (5.3%) | 9 (34.6%) | <0.001 |
| Congenital renal dysplasia | 2 (3.6%) | 0 | NS |
| Other | 11 (19.7%) | 4 (15.4%) | NS |

BW, body weight; DBD, donation after brain death; DBCD, donation after brain and cardiac death; DCD, donation after cardiac death; FSGS, Focal segmental glomerulosclerosis; HD, hemodialysis; PD, peritoneal dialysis;

NS, not significant, P>0.05
